# The role of telocytes in morphogenetic bioelectrical signaling: once more unto the breach

**DOI:** 10.3389/fnmol.2014.00041

**Published:** 2014-05-13

**Authors:** Lawrence Edelstein, John Smythies

**Affiliations:** ^1^Medimark CorporationDel Mar, CA, USA; ^2^Department of Psychology, Center for Brain and Cognition, University of CaliforniaSan Diego, La Jolla, CA, USA

**Keywords:** morphogenetic code, bioelectrical signaling, telocytes, gap junctions, information processing, Vmems, network dynamics, stem cells

## Introduction

This paper explores the possibility that telocytes play a major role in morphogenetic bioelectrical signaling.

## The facts

In a series of recent papers, Levin and his coworkers (Levin, 2009, 2012; Adams and Levin, 2012; Tseng and Levin, 2012, 2013) introduced the concept of a morphogenetic code based on bioelectrical signaling between cells. They showed that patterns of resting potentials (Vmem) in non-excitable cells act as instructive signals during embryogenesis, regeneration and cancer suppression in a wide range of tissues. They state that bioelectrical signaling among non-excitable cells, coupled by gap junctions, simulates neural network-like dynamics and underlies the information-processing functions required by complex pattern formation *in vivo* which result in coherent changes in anatomy. They identified one intermediate step between a specific bioelectric signal and altered cell anatomy as the regulation of small signaling molecules through transporters. Furthermore, they showed that the particular morphological change induced is dependent only on the voltage itself and not on the genetic identity of the channel, nor on the chemical species of the ion involved. Evidence from embryology suggests that narrow ranges of transmembrane potential specify the growth of specific anatomical structures such as an eye, head or tail. The authors suggest that this system may form a code, given that each cell has not one but many domains of Vmem along its surface, and so the spatial distribution of voltage values could form a rich combinatorial code. Vmem gradients form an important signal for modulating stem cell proliferation and differentiation (Pai and Levin, 2014). The authors conclude “… our hypothesis is that the existing highly successful theoretical apparatus for information processing in neurobiology could be extended to understand the properties of highly dynamic, self-repairing tissues and organs… we wondered whether in some cases, the bioelectric signal also activates a positional information pathway that guides the induced growth toward the locally-appropriate anatomical identity.”

## Our hypothesis

We will present the case that telocytes (TCs) may play an essential role in morphogenetic bioelectrical signaling. In previous papers we have reviewed the key role that TCs may play in morphogenesis in nearly all organs in the body (Smythies and Edelstein, 2013; Edelstein and Smythies, 2014). Telocytes are characterized by having very small cell bodies consisting of a nucleus and a small amount of cytoplasm, and “extremely long and thin” tubular processes called telopodes up to 100 micrometers long, yet only 20–200 nanometers wide (Popescu and Faussone-Pellegrini, 2010; for the most comprehensive review of the literature see www.telocytes.com). The caliber of the telopodes is not uniform, which possess very thin podomers and dilations named podoms (Gherghiceanu and Popescu, 2012). They make synapses of various kinds, including adhaerens contacts and gap junctions, with practically every other type of cell in the tissue, including blood vessels, nerve fibers, fibroblasts, muscle cells, immune cells, glandular cells, stem cells and also other TCs. Thus, they form a continuous net wrapped around the larger cells of the organ. They exhibit a wide range of functions. The present consensus is that TCs could form an extensive intercellular information transmission and executive system that may utilize electric currents, small molecules, exosomes—and possibly electrical events within the cytoskeleton—to modulate homeostasis, stem cell activity, tissue repair, peristalsis, anticancer activity and other complex functions in many organs (Gherghiceanu and Popescu, 2010; Popescu et al., 2011; Cretoiu et al., 2012a,b,c, 2013; Popescu et al., 2012; Luesma et al., 2013). These activities include extensive morphogenetic tissue repair after injuries such as cardiac ischemia and stroke in which the recruitment of stem cells plays a major role (Manole et al., 2011; Zhao et al., 2013).

Telocytes are also involved in the electrical modulation of excitable tissue, such as the smooth muscle of the gut and uterus. They are reported as being capable of spontaneously initiating electrical activity, which can be modulated by the stimulation of enteric nerves (Zhu et al., 2013); this process involves calcium transients (Yamashita, 2010). Cretoiu et al. (2013) reported the existence of hyperpolarization-activated chloride-inward current with calcium dependence and the absence of L-type calcium channels in human myometrium. Small-conductance calcium-activated potassium (SK3) channels have been detected in TCs in human myometrium wherein they reduce contractility (Rosenbaum et al., 2012). There is also evidence that they may mediate cholinergic neuromuscular transmission in ileal smooth muscle (Tanahashi et al., 2013). Sheng et al. (2014) examined human cardiac-cultured TCs and found that these cells expressed large conductance Ca(2+) -activated K(+) current (BKCa) and inwardly rectifying K(+) current (IKir), but not transient outward K(+) current (Ito) and ATP-sensitive potassium current (KATP). Other TC functions include modulation of glandular and immune activity. The reader is referred to our previous papers for further details.

We suggest that the TC system is well equipped to form a major part of the bioelectric “information pathway” postulated by Levin (2012) between cells in most tissues. The system may operate as follows: Since the TC net makes gap junction contacts with the cells in an organ its electrical activity could modulate, and be modulated by, the individual Vmems of these cells. Furthermore, the TC net could support wide-reaching patterns of the network dynamics postulated by Levin (2012). This in turn could modulate all of the other functions exerted by the TC net including morphogenesis, cancer control, as well as tissue repair and remodeling functions. In a recent review of gap junctions, Shimizu and Stopfer (2013) stated that their properties can be complex and surprising. Gap junctions help generate, propagate, and regulate neural oscillations, filter electrical signals, and can be modulated in a variety of ways. However, it is possible that a system of information transfer “A” between cells, which depends only upon direct sequential cell-to-cell gap junctions, might be less efficient than a system “B” such as the TC net, which is wholly devoted to the broad and rapid transmission of information via gap junctions. Furthermore, the fast integration of Vmem information with other sources of - and targets for - this information would be harder for “A” than for “B.” As well, the TC net provides a mechanism for the immediate coordinated recruitment of stem cells that system “A” so far lacks.

We therefore posit that the universally distributed, electrically active, gap-junction linked, morphogenetic, cancer-suppressing and stem cell-controlling TC network supplements, but does not replace, direct cell-to-cell systems in bioelectrical signaling.

### Conflict of interest statement

The authors declare that the research was conducted in the absence of any commercial or financial relationships that could be construed as a potential conflict of interest.
